# Long-term outcomes of ciliary sulcus versus capsular bag fixation of intraocular lenses in children: An ultrasound biomicroscopy study

**DOI:** 10.1371/journal.pone.0172979

**Published:** 2017-03-16

**Authors:** Yun-e Zhao, Xian-hui Gong, Xue-ning Zhu, He-ming Li, Meng-jun Tu, Terry G. Coursey, Stephen C. Pflugfelder, Feng Gu, Ding Chen

**Affiliations:** 1 Eye Hospital, Wenzhou Medical University, Wenzhou, Zhejiang, China; 2 Cullen Eye Institute, Department of Ophthalmology, Baylor College of Medicine, Houston, Texas, United States of America; Bascom Palmer Eye Institute, UNITED STATES

## Abstract

**Purpose:**

To evaluate the long-term outcomes of ciliary sulcus versus capsular bag fixation of intraocular lenses (IOLs) in children after pediatric cataract surgery.

**Methods:**

IOL was implanted in the ciliary sulcus in 21 eyes of 14 children, and in the capsular bag in 19 eyes of 12 children for the treatment of pediatric cataract in an institutional setting. Ultrasound biomicroscopy (UBM) was performed. Main outcome measures included IOL decentration, IOL tilt, anterior chamber depth (ACD), angle-opening distance at 500 μm (AOD_500_), trabecular-iris angle (TIA), best-corrected visual acuity (BCVA), intraocular pressure (IOP), and incidence of postoperative complications.

**Results:**

The mean follow-up period was 6.81 ± 1.82 years. Comparing to the capsular bag fixation group, the ciliary sulcus fixation group had higher vertical IOL decentration, horizontal IOL tilt, and vertical IOL tilt (*p* = 0.02, 0.01,0.01, respectively), higher incidence of iris-IOL contact and peripheral anterior synechia (*p* = 0.001, 0.03, respectively), smaller ACD, AOD_500_, and TIA (*p* = 0.02, 0.03, 0.04, respectively), higher mean IOP (17.10 ±6.06 mmHg vs.14.15± 4.74 mmHg, *p* = 0.01), and higher incidence of secondary glaucoma (28.57% vs. 10.53%, *p* = 0.007).There was no significant difference between the two groups with regard to the BCVA, refractive errors, incidence of myopic shift, nystagmus, strabismus, and visual axis opacity.

**Conclusions:**

Ciliary sulcus fixation of IOLs in pediatric eyes may increase IOL malposition and crowding of the anterior segment, and may associate with a higher risk of secondary glaucoma compared to capsular bag fixation of IOLs.

## Introduction

Pediatric cataracts are a major cause of childhood blindness affecting approximately 200,000 children worldwide, with an estimated prevalence ranging from three to six per 10,000 live births [1–3]. Depending on the etiology and age of onset, pediatric cataracts can be either congenital (which usually occurs before infancy) or acquired (developed after infancy). Over the past two decades, advances in surgical equipment and techniques have considerably improved the outcome of pediatric cataract surgery. Intraocular lens (IOL) implantation in the pediatric cases is now a generally accepted alternative form of optical correction to contact lenses and spectacles [4].

A posterior chamber IOL (PCIOL) inserted into the capsular bag is always preferred during cataract surgery. However, in certain circumstances, the PCIOL is placed in the ciliary sulcus in the case of intraoperative posterior capsule rupture, rupture of the zonules of Zinn, or secondary IOL implantation [5]. The safety and effect of ciliary sulcus fixation of PCIOL has been well established and accepted in adult patients if the remaining lens capsule is sufficient and strong enough to secure the IOL [6,7]. However, little is known regarding the long-term outcome and safety of ciliary sulcus fixation of IOL in pediatric cases. It is well understood that a child’s eye differs significantly from the adult eye in size and changing axial length and corneal curvature over a period of time [8]. Additionally, children have a much longer life span after cataract removal and IOL implantation.

In this study, we utilized ultrasound biomicroscopy (UBM) to assess the characteristics of the anterior segment and IOL position in children who underwent pediatric cataract surgeries and PCIOL implantation with a follow-up period of more than five years. Our major purpose was to compare the long-term outcomes of ciliary sulcus versus capsular bag fixation of IOLs in pediatric pseudophakias.

## Patients and methods

This was a retrospective, comparative observational case series. Children with pediatric cataract underwent cataract surgery and PCIOL implantation at the Eye Hospital of Wenzhou Medical University from January 2005 to December 2009 and followed up for at least five years.

The medical records of all patients were reviewed. Patients with microcornea (horizontal diameter < 9.0 mm) or with other primary disease (e.g., chronic anterior uveitis, trauma, anterior segment dysgenesis, optic nerve or other fundus abnormalities, prematurity and cataract associated with other syndromes, maternal rubella syndrome, and systemic disorders) were excluded. Patients with signs of congenital glaucoma before surgery, including increased intraocular pressure (IOP), along with increased corneal diameter and corneal edema, were also excluded. Eligible patients were categorized into two groups. The study group was comprised of eyes with IOL implanted in ciliary sulcus, and the control group was comprised of eyes with IOL implanted in the capsular bag.

Ethical approval for this study was provided by the Ethics Committee of the Eye Hospital of Wenzhou Medical University. All measurements followed the tenets of the Declaration of Helsinki, and written informed consents were obtained from the legal guardians of the minor subjects enrolled in the study.

### Surgical technique

All surgeries were performed by one experienced surgeon (Y.E.Z) under general anesthesia. There was some variation in surgical technique depending on the age of the patient and the year of surgery. The youngest patients were treated with a pars plana or limbal lensectomy and anterior vitrectomy with care taken to remove as much lens matter as possible. The remaining patients were treated with phacoemulsification and aspiration with or without primary posterior capsulotomy and anterior vitrectomy. Patients underwent primary IOL implantation at the time of lens removal, or postponed secondary IOL implantation after a period of spectacle use after lens removal, depending on the age and characteristics of patient at surgery. PCIOL was placed in the ciliary sulcus in cases of intraoperative posterior capsule defect or rupture, or failing to open the capsular bag during the secondary IOL implantation. The IOLs used for ciliary sulcus fixation were all foldable 3-pieces hydrophobic acrylic IOL (Acrysof; Alcon Laboratories Inc., Fort Worth, TX, USA) with model of MA60AC or MA60BM. All IOLs implanted in the capsular bag in the control group were foldable 1-piece hydrophobic acrylic IOL (Acrysof; Alcon Laboratories Inc., Fort Worth, TX, USA) with model of SA60AT or SN60AT. All IOLs had the haptics oriented in the horizontal axis when implanted.

After surgery, eyes were treated with topical antibiotics, corticosteroids, NSAID, and mydriatic and cycloplegic agents. The use of potent corticosteroids eye drops, such as prednisolone acetate 1%, was initiated and tapered over time or replaced with less potent drops, such as loteprednol etabonate 0.5%, to lower the incidence of steroid-induced ocular hypertension. Eye patching for three to six hours a day was prescribed immediately after surgery to manage amblyopia.

### Postoperative follow up and ophthalmic examinations

After surgery patients were followed up on a regular basis at our hospital. The best-corrected visual acuity (BCVA) was assessed using a Snellen chart with the patient wearing optimal refractive correction. The power of the spectacles was determined by retinoscopy. The refractive error was calculated as the spherical equivalent (SE). Strabismus was evaluated with Krimsky test or alternative cover test. IOP was evaluated with Perkins handheld applanation tonometer under anesthesia. Indirect ophthalmoscopy was used to examine the fundus after dilation. Any postoperative complications were recorded. Secondary glaucoma was diagnosed according to the criteria suggested by Infant Aphakia Treatment Study [9].

### Ultrasound biomicroscopy of the ocular anterior segment

Ultrasound biomicroscopy examination was performed for all subjects using the OTI Scan 3000 (Optos Hialeah, Hialeah, FL, USA). Scanning was conducted with the non-dilated eye in a central position and the patient in the supine position. A drop of a topical anesthetic agent (benoxinate hydrochloride) was applied to both eyes. A cup suitable to the patient's palpebral fissure size was then inserted between the lids. Sterile normal saline solution was used to fill the cup to the appropriate level. The UBM probe with a 35 MHz tip was used to obtain four radial scans at 12:00 o'clock and 3:00 o'clock while kept perpendicular to the ocular surface and central in the pupil. The depth of scans ranged from the anterior corneal surface to the posterior lens capsule. Clips of the scans of each eye were reviewed and the best quality frames were chosen for data acquisition. The UMB images were exported and analyzed with Image-Pro Plus version 7 (Media Cybernetics, Rockville, MD, USA).

**Fig 1:**
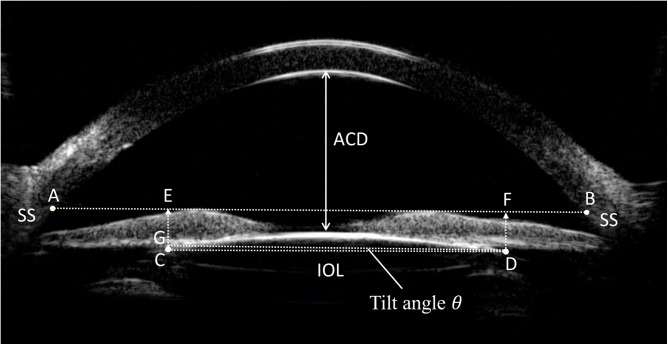
Measurement of the anterior chamber depth (ACD) and intraocular lens (IOL) decentration and tilt on the ultrasound biomicroscopy image of the anterior segment. ACD was determined from the central inner corneal surface, perpendicular to the corneal surface to the most anteriorly visible part of the IOL. A line (line AB) was drawn between the two scleral spurs (SSs), as the base line of reference for IOL position. Two perpendicular lines were drawn from both optical endpoints of the IOL (C and D) to the base line with intersection points (E and F). IOL decentration was equal to half of the differences between distance AE and FB, i.e. IOL decentration = (|AE—FB|) / 2. IOL tilt was determined by the angle (*θ*) formed by the line between the two optical endpoints and the base line. A line parallel to line AB was drawn intersecting one of the optical endpoints (D). Angle θ was calculated with following formula:
$$\theta =\arctan(\frac{CG}{DG})\times \frac{180}{\pi }=\arctan(\frac{\left|CE-DF\right|}{EF})\times \frac{180}{\pi }$$

**Fig 2:**
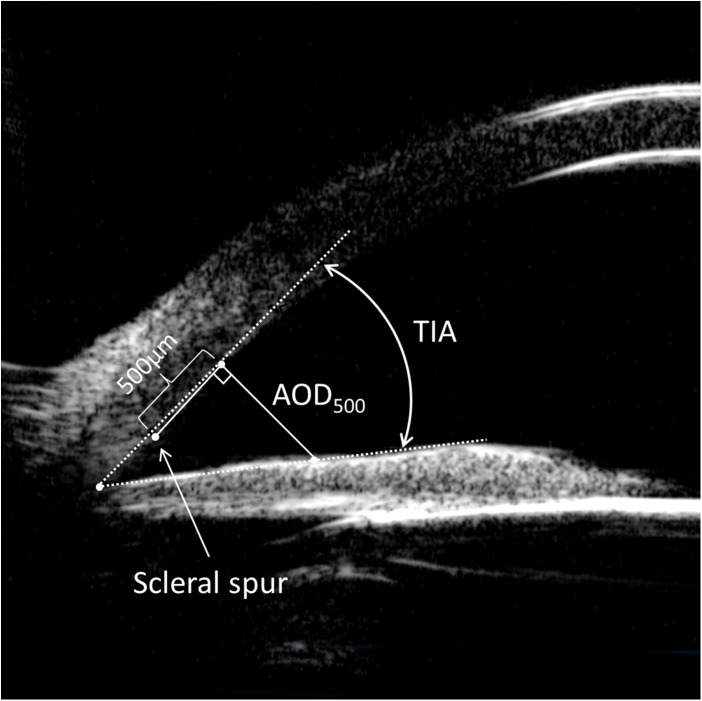
Measurement of the angle on the ultrasound biomicroscopy image of the anterior segment. Angle-opening distance at 500 μm (AOD_500_) was measured on a line perpendicular to the trabecular meshwork at points 500 μm from the scleral spur. Trabecular-iris angle (TIA) was measured with the apex in the iris recess and the arms of the angle passing through a point on the trabecular meshwork 500 μm from the scleral spur and the point on the iris perpendicularly opposite.

The scleral spur (SS) showed a high echo area that resembles an olecranon (elbow shape) in the UBM image. The line between the two olecranons was considered as the base line of reference for IOL position. For IOL decentration, two perpendicular lines were drawn from both optical endpoints of the IOL to the base line and the distances between intersection points and the SS were measured. IOL decentration was equal to half of the differences between these two distances (Fig 1). IOL tilt was determined by the angle (*θ*) formed by the line between the two optical endpoints and the base line. The tilting angle (*θ*) was calculated by arctan function: $\theta =\arctan(\frac{CG}{DG})\times \frac{180}{\pi }=\arctan(\frac{\left|CE-DF\right|}{EF})\times \frac{180}{\pi }$, (Fig 1). The IOL decentration and tilt were calculated in both horizontal and vertical directions. Anterior chamber depth (ACD) was determined from the central inner corneal surface, perpendicular to the corneal surface to the most anteriorly visible part of the IOL (Fig 1). This angle was evaluated using the angle-opening distance at 500 μm (AOD_500_) and the trabecular-iris angle (TIA), as proposed by Pavlin and associates [10]. AOD_500_ was measured on a line perpendicular to the trabecular meshwork at points 500 μm from the SS, and TIA was measured with the apex in the iris recess and the arms of the angle passing through a point on the trabecular meshwork 500 μm from the scleral spur and the point on the iris perpendicularly opposite (Fig 2). All four quadrant values were obtained and averaged for statistical analysis. Any abnormities in the configuration of the anterior segment structure including the angle, iris, ciliary body, capsule, and IOL was documented.

### Statistical analysis

Statistical analysis was performed using SPSS for Windows software version 17.0 (SPSS Inc., Chicago, IL, USA). All data was analyzed for normality using the Kolmogorov-Smirnov test, revealing several instances of non-normal distributions, which recommended non-parametric statistical analyses. Values are expressed as mean ± standard deviation (SD) (range) or median (range). Independent sample t-test, Mann-Whitney test, Pearson chi-square test, or Fisher's exact test was used for statistical analysis as appropriate. A *p*-value < .05 was considered statistically significant.

## Results

Forty eyes of 26 children underwent pediatric cataract surgery and PCIOL implantation met our criteria for inclusion. PCIOL was implanted in ciliary sulcus in 21 eyes of 14 children. The indications of ciliary sulcus fixation of IOL were intraoperative posterior capsule defect/rupture in 5 eyes and failure to open the capsular bag during the secondary IOL implantation in 16 eyes. PCIOL was implanted in capsular bag in 19 eyes of 12 children. Preoperative demographics and clinical characteristics are summarized in Table 1. The mean follow-up period was 6.81 ± 1.82 years (range, 5.17–11.20 years). There were no significant differences between the two groups with regard to the gender, type of cataract, age at lens removal, age at IOL implantation, follow-up duration, age at last visit, and preoperative IOP.

**Table 1:** Demographics and clinical characteristics of children underwent pediatric cataract surgery and posterior chamber intraocular lenses implantation.

	Ciliary sulcus fixation	Capsular bag fixation	*p*
No. of eyes/patients	21/14	19/12	-
Sex, No. (%) of female	6 (42.86%)	5 (41.67%)	0.5
Type of cataract, Congenital vs. Acquired	10/11	7/12	0.1
Age at lens removal, years (range)	2.10± 2.05 (0.17–5.33)	3.26±1.98 (1.08–5.83)	0.08
Age at IOL implantation, years (range)	3.85± 2.46 (1.25–6.25)	4.53±2.92 (1.42–8.08)	0.2
Primary vs. secondary IOL implantation, eyes	5/16	13/6	0.01
Follow up duration, years (range)	7.18 ±1.85 (5.17–11.20)	6.94 ±1.69 (5.33–10.17)	0.5
Age at last visit, years (range)	11.03± 2.46 (7.92–15.25)	11.47±3.12 (6.83–16.08)	0.6
Preoperative IOP, mmHg (range)	10.26 ± 2.25 (7.64–15.18)	11.12 ± 3.05 (7.42–16.35)	0.4

IOL = intraocular lenses, IOP = intraocular pressure.

**Fig 3:**
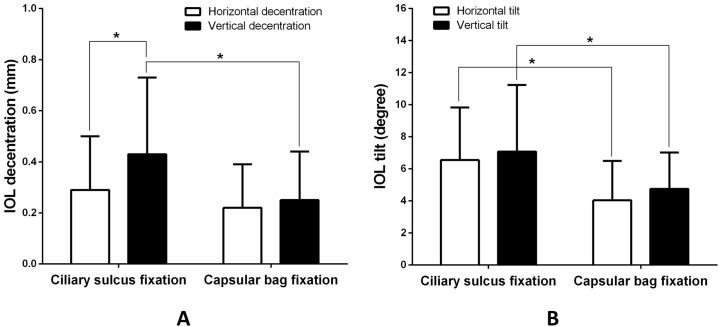
**Comparison of intraocular lens (IOL) decentration (A) and IOL tilt (B) between the ciliary sulcus fixation and capsular bag fixation groups in horizontal and vertical directions**. (* indicates significant difference groups, *p* < 0.05)

The outcomes of ultrasound biomicroscopy of the anterior segment are listed in Table 2. The mean value of IOL decentration was significantly higher in the vertical than in the horizontal direction in the ciliary sulcus fixation group (*p* = 0.03). There was significant difference between the ciliary sulcus fixation and capsular bag fixation groups with regard to the vertical IOL decentration, horizontal IOL tilt, and vertical IOL tilt (*p* = 0.02, 0.01, 0.01, respectively), but not horizontal IOL decentration (Table 2 and Fig 3). In cases of significant IOL tilt, residual lens material with corticohyperplassia was observed in four eyes in the sulcus fixation group and one eye in the capsular bag fixation group, and enlarged Soemmering's ring was found in two eyes in the sulcus fixation group. IOL subluxation was observed in one eye in the ciliary sulcus fixation group, due to insufficient support of the residual capsule with one haptic fixated at the sulcus and the other haptic at the ciliary body. Surgery with the scleral fixation technique was performed to reposition the IOL.

**Table 2:** Outcomes of ultrasound biomicroscopy of the anterior segment in children underwent ciliary sulcus versus capsular bag fixation of posterior chamber intraocular lenses.

	Ciliary sulcus fixation (n = 21)	Capsular bag fixation (n = 19)	*p*
Horizontal IOL decentration (mm)	0.29 ±0.21 (0.05–0.66)	0.22 ±0.17 (0.03–0.47)	0.15
Vertical IOL decentration(mm)	0.43 ±0.30 (0.08–0.98) ^a^	0.25 ±0.19 (0.02–0.59)	0.02
Horizontal IOL tilt (degree)	6.55 ±3.28 (1.18–10.71)	4.04 ±2.45 (1.03–7.24)	0.01
Vertical IOL tilt (degree)	7.08±4.16 (1.52–12.56)	4.75±2.27 (1.21–8.13)	0.01
ACD(mm)	3.75 ± 0.74 (2.62–4.59)	4.28 ± 0.52 (3.19–4.92)	0.02
AOD_500_ (μm)	445.84 ±65.20 (348–517)	471.15 ±56.97 (394–538)	0.03
TIA (degree)	45.28 ±8.52 (28.43–57.98)	51.66 ±8.38 (34.87–60.12)	0.04
Iris-IOL contact (No. (%))	13 (61.90%)	1 (5.26%)	0.001
Peripheral anterior synechia (No. (%))	6 (28.57%)	2 (10.53%)	0.03
Residual lens material (No. (%))	4 (19.05%)	1 (5.26%)	0.001
Enlarged Soemmering's ring (No. (%))	2 (9.52%)	0 (0%)	< 0.001

IOL = intraocular lens, ACD = Anterior chamber depth, AOD_500_ = angle-opening distance at 500 μm, TIA = trabecular-iris angle

^a^ for significant difference between vertical and horizontal IOL decentration.

The mean ACD in the ciliary sulcus fixation group (3.75 ± 0.74 mm) was significantly shallower than the capsular bag fixation group (4.28 ± 0.52) (*p* = 0.02). The mean AOD_500_ and TIA of pseudophakias in the ciliary sulcus fixation group were significantly smaller than the capsular bag fixation group (*p* = 0.03, 0.04, respectively) (Table 2). Thirteen eyes (61.90%) in the ciliary sulcus fixation group presented iris-IOL contact, among which, eight eyes had iris-optic contact in the midperiphery of the IOL, while the other five eyes had iris-haptic contact. IOL forward shifting with optic and haptics embedding into iris tissue was observed in one eye diagnosed with angle-closure glaucoma (ACG). Only one eye showed iris-IOL contact in the capsular bag fixation group. Peripheral anterior synechia was more frequently seen in the sulcus fixation group than in the capsular bag fixation group (28.75% vs. 10.53%, *p* = 0.03). Most synechiae were localized and did not exceed two quadrants and were close to the quadrants where the surgical incisions were made.

The visual outcomes at last visit and postoperative complications of both groups are listed in Table 3. Overall, 85% of patients achieved a final BCVA of 20/50 or better. No significant differences were present between the two groups with regard to the BCVA, refractive error, and incidence of myopic shift, nystagmus, strabismus and visual axis opacity (all *p* > 0.05). The mean IOP of the ciliary sulcus fixation group was 17.10 ± 6.06 mmHg (range, 11.30–30.58 mmHg), which was significantly higher than the capsular bag fixation group (14.15± 4.74 mmHg, range, 9.36–23.97 mmHg) (*p* = 0.01). In the ciliary sulcus fixation group, six eyes were diagnosed with secondary glaucoma, including four eyes with open-angle glaucoma (OAG) and two eyes with ACG. In the capsular bag fixation group, two eyes were diagnosed with OAG. The incidence of secondary glaucoma in the ciliary sulcus fixation group (28.57%) was significantly higher than the capsular bag fixation group (10.53%) (*p* < 0.01). Surgical removal of a pupillary membrane and peripheral iridectomy were performed to control IOP in the two eyes with ACG. All other eyes with OAG were treated with anti-glaucoma medications.

**Table 3:** Long-term visual outcomes and postoperative complications of ciliary sulcus versus capsular bag fixation of posterior chamber intraocular lenses in children.

	Ciliary sulcus fixation (n = 21)	Capsular bag fixation (n = 19)	*p*
BCVA	20/50 (20/200–20/20)	20/40 (20/300–20/15)	0.29
Refractive error (SE), D	−2.19±2.25 (−6.00 - +1.63)	−1.87±2.13 (−4.63 - +1.50)	0.18
Myopic shift (No. (%))	13 (61.90%)	11 (57.89%)	0.22
Nystagmus (No. (%))	4 (19.05%)	4 (21.05%)	0.26
Strabismus (No. (%))	5 (23.81%)	3 (15.79%)	0.12
Visual axis opacification (No. (%))	2 (9.52%)	3 (14.29%)	0.09
Secondary glaucoma (No. (%)	6 (28.57%)	2 (10.53%)	0.007

BCVA = best-corrected visual acuity, SE = spherical equivalent, D = diopter

## Discussion

The feasibility of IOL implantation in pediatric eyes has long been established [4]. The major advantage of an IOL is that it provides permanent continuous correction of aphakia, which is important in preventing amblyopia and fostering normal visual development in young children [11]. Placement of a posterior chamber IOL into the capsular bag is preferred as it results in a close-to-normal position. In some cases (i.e., intraoperative posterior capsule rupture, or inability to open the capsular bag during the secondary IOL implantation), the IOL is inserted into the ciliary sulcus with the premise of sufficient support of the remaining lens capsule [5]. Ciliary sulcus fixation of IOL in pediatric eyes has been proven to be reasonably safe and effective over the short-term according to several studies [12–14]. However, the pediatric eye is still developing and changes rapidly over a period of time, and the data on the long-term outcome of IOL implantation in ciliary sulcus in children is limited. In this study, we evaluated the anterior segment including the status of IOL with UBM in pediatric pseudophakias with a follow-up period ranging from 5 to 11 years. To our knowledge, this is the first reported comparative study of the long-term outcomes of the ciliary sulcus fixation vs. capsular bag fixation of IOLs in pediatric eyes with UBM.

In the present study, the amount of IOL decentration and tilt measured by UBM in the sulcus fixation group appears generally larger than the capsular bag fixation group. In particular, the vertical IOL decentration and tilt in sulcus fixation pseudophakic eyes was 0.43 ± 0.30 mm and 7.68±5.16 degrees, with a significant standard deviation, and beyond the 0.4 mm limit of decentration and 7 degrees limit of tilt for tolerable aberration induced by IOL malposition, as proposed by Holladay and associates [15]. This suggests that ciliary sulcus fixation is more prone to IOL malposition comparing to the capsular bag fixation, which is consistent with previous reports [16–18]. A UBM study performed by Loya and associates [16] reported that more than half the IOLs implanted in the ciliary sulcus were not in the intended location. Sauer and Mester [17,18] evaluated the IOL position using a Purkinjemeter and found the decentration and tilt of sulcus-fixated IOLs were much higher than those of capsular bag fixated IOLs, which also exceeded the tolerable amounts derives from eye model experiments.

IOL malposition may be the result of the original surgical placement of the lens, or it may develop postoperatively because of external or internal factors. External factors include trauma or eye rubbing [19]. None of the patients in our study had a positive history of trauma suggesting that internal factors may have a significant role in IOL malposition. Interestingly, we found the horizontal IOL decentration in ciliary sulcus fixation group appears within the limit suggested by Holladay and associates [15], and did not differ significantly from the capsular bag fixation group. We hypothesize that the size disparity between the IOL and ciliary sulcus, and gravity may account for a greater tendency for vertical decentration than horizontal decentration. Trivedi and associates [20] found that longer eyes, associated with wider sulcus-to-sulcus distance, may promote IOL decentration. We did not measure the axial length or the width of ciliary sulcus to confirm this. In addition, residual lens material and/or enlarged Soemmering's ring was detected by UBM in a few cases of significant IOL tilt in our study. Asymmetry in ciliary sulcus caused by these abnormalities may lead to the asymmetry in the position of IOL. Other internal factors contributing to IOL malposition may include scarring, synechiae formation, and capsular contraction [20]. Most patients with IOL malposition in our study were managed conservatively. Only one pseudophakia with subluxation of the 3-piece IOL required surgical repositioning. The rate of surgical repositioning is comparable to the finding reported by Trivedi and associates (5.2%) [20].

Implantation of the IOL in the ciliary sulcus tends to crowd the anterior segment in pediatric eyes. In general, the ACD, AOD_500_ and TIA of the pseudophakias in ciliary sulcus fixation group were smaller than those in capsular bag fixation group in our study. The haptics of more anteriorly located IOLs in ciliary sulcus may push the peripheral iris slightly forward, leading to a narrower angle dimension, as indicated by smaller AOD_500_ and TIA. The incidence of iris-IOL contact in the ciliary sulcus fixation group (61.90%) was much higher than the capsular bag fixation group. The contact between the optic and/or haptics of IOL and the uveal tissues increases the risk of iris chaffing and contributes to postoperative inflammation [21,22]. While the acute postoperative inflammation is mainly affected by the extent of the tissue damage during the surgery complicated with iris damage, posterior lens capsule rupture and vitreous loss, the chronic postoperative inflammation has been attributed to rubbing of iris and IOL [22–24]. Amino and associates [22] detected anterior chamber flare with ciliary sulcus IOL fixation more than two years after surgery. The long-term comparison of inflammation in anterior chamber between the ciliary sulcus fixation and capsular bag fixation groups was not considered in our study. However, the higher incidence of peripheral anterior synechia in the ciliary sulcus fixation group may indicate a greater risk for postoperative inflammation. In addition, the narrower angle dimension of this group makes development of peripheral anterior synechia formation more likely.

Development of secondary glaucoma is a major sight-threatening complication after pediatric cataract surgery [25–29]. The incidence of secondary glaucoma varies in different reports, ranging from 10% to 37% [9, 25–30]. The incidence increases if patients are followed up for a longer period. The Infant Aphakia Treatment Study (IATS) reported an incidence of 8.8% of glaucoma at the one-year follow-up visit [9]. The incidence of glaucoma of the same cohort increased to 17.0% in the five-year report [25]. In our study, the long-term incidence of secondary glaucoma was 20.0% in all cases. The incidence in the ciliary sulcus fixation group was higher than the capsular bag fixation group (28.6% vs. 10.5%). This finding is consistent with previous reports [14,31]. As previously stated, ciliary sulcus fixation of IOL may cause crowding of the anterior segment and increase the risk of postoperative inflammation, leading to synechia formation in the chamber angle, pupillary block, and iris bombé which triggers angle-closure glaucoma [32]. In the two eyes with angle-closure glaucoma, narrowing angle with peripheral anterior synechia was seen in three quadrants. One of these had a forward shifting IOL with optic and haptics embedding into the iris tissue. These old signs of synechiae and pupillary block do not seem to regress with time, even after glaucoma treatment, indicating the underlying etiology of this type of glaucoma. Open angle glaucoma accounts for a majority of the long-term secondary glaucoma after pediatric cataract surgery [25,26,29]. However, the underlying etiology of secondary open angle glaucoma remains undetermined. Numerous risk factors have been suggested, including younger age at detection of cataract, small corneal diameters, coexistence of persistent fetal vasculature (PFV), a family history of aphakic glaucoma, surgery in the first year of life, and primary posterior capsulotomy/anterior vitrectomy performed at the time of cataract surgery [9,29,30,33]. We speculate that postoperative inflammation may compromise the trabecular meshwork and contribute to the development of secondary open angle glaucoma. Further investigation is warranted to confirm this hypothesis.

The aim of pediatric cataract treatment is to achieve good visual function for the life of the child. With proper postoperative care and amblyopia therapy, the visual outcomes of most pediatric cataract surgery are satisfactory [34–36]. In our study, 85% of patients achieved a final BCVA of 20/50 or better after more than five years of follow up. Although there was dramatic improvement in VA in some cases, most patients had amblyopia and the overall level of VA was far from normal values. Those with nystagmus and presbyopia tended to have poorer visual prognosis. Interestingly, there was no significant difference in the final visual outcomes between the ciliary sulcus fixation and capsular bag fixation groups. The visual outcome in our study is comparable to the previous reports [34–36]. However, it is difficult to compare the visual outcomes of pediatric cataract surgery between different groups or different studies because of general limitations, including variable age at surgery, varying surgical technique, variation in associated ocular pathology, and variable compliance to amblyopia therapy [26,34–39]. Visual axis opacity (VAO) used to be the most common cause of poor postoperative visual acuity [35,40]. The incidence of VAO was much lower in our study than that in the literature [35,40], and no significant difference was found between the two groups. This is probably due to the high rate of posterior capsulotomy and anterior vitrectomy performed in our study [41].

Limitations of this study include the retrospective design and the relatively small sample size. Also, the effect of the variation in surgical techniques and the types of IOL (3-piece IOL vs. single-piece IOL) on the outcome was not taken into consideration. Major strengths of this study include: all procedures were performed by the same experienced surgeon, long-term follow up for more than five years, and detailed ultrasound biomicroscopic analysis of the anterior segment in pediatric pseudophakia.

## Conclusion

Fixation of IOLs in the ciliary sulcus increases IOL malposition and crowding of the anterior segment in pediatric eyes, and is associated with higher incidence of secondary glaucoma. Nonetheless, the long-term visual outcome is comparable to the capsular fixation of IOLs. Ultrasound biomicroscopy is a useful tool in evaluating the anterior segment of the pediatric pseudophakias and is recommended for long-term follow up.
